# Surface Tensions of Alkyl Lactates with *n*-Alkanols or Branched Alkanols

**DOI:** 10.1021/acs.jced.2c00629

**Published:** 2022-12-28

**Authors:** Carmen Almodovar, Héctor Artigas, Saoussen Wacharine, Kaïs Antar, Carlos Lafuente

**Affiliations:** † Departamento de Química Física, Facultad de Ciencias, Universidad de Zaragoza, Zaragoza50009, Spain; ‡ Faculté des Sciences, Laboratoire des Matériaux, Cristallochimie et Thermodynamique Appliquée, LR15ES01, Département de Chimie, Université de Tunis EL Manar, Tunis2092, Tunisia

## Abstract

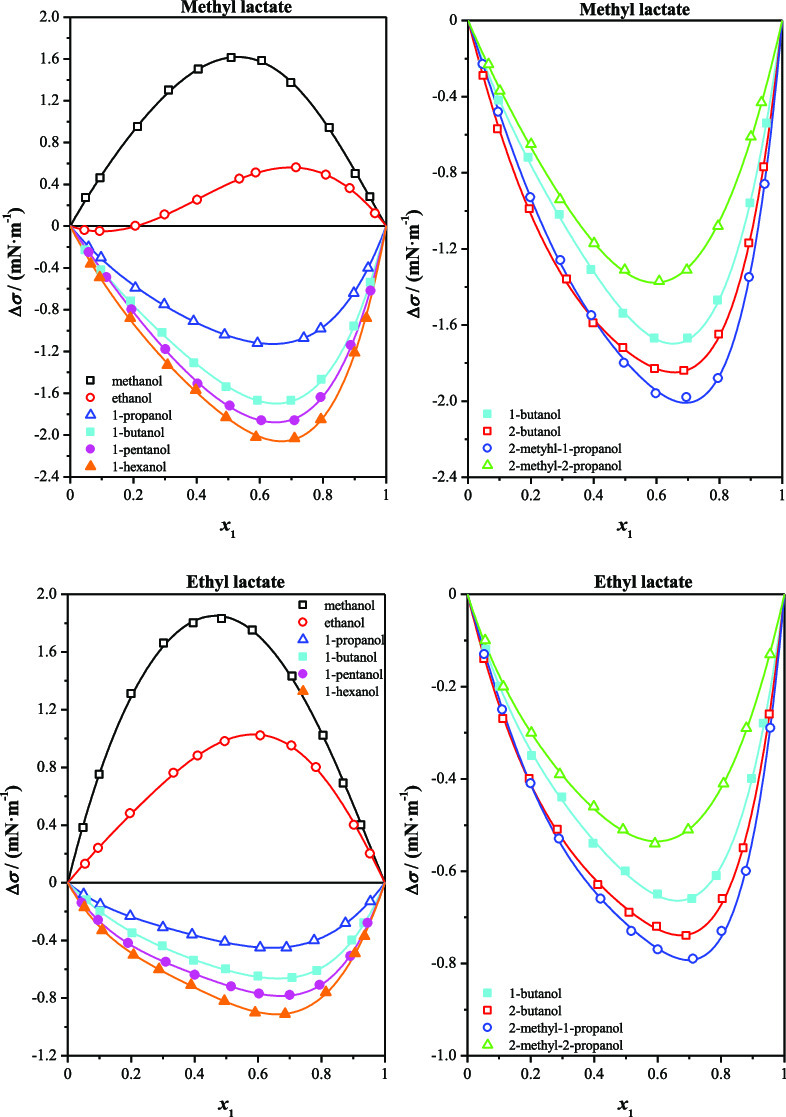

The surface behavior of 18 binary mixtures at *T* = 298.15 K and at atmospheric pressure (*p* = 0.1 MPa) was measured with a drop volume tensiometer. These mixtures are formed by alkyl lactates (methyl lactate and ethyl lactate) and alcohols (*n*-alkanols from methanol to 1-hexanol and isomeric butanols). Surface tension deviations for all these systems were calculated and correlated with the mole fraction using the Myers–Scott equation. For the mixtures alkyl lactate with methanol, the surface tension deviations are positive. With regard to the mixtures containing ethanol, the surface tension deviation is positive for methyl lactate and slightly negative in the lactate-poor region for ethyl lactate. For the rest of the mixtures, the surface tension deviations are negative. A molecular interpretation of the different behaviors observed was proposed.

## 1 Introduction

The quest for greener chemical processes in the society induces all around the world strict environmental regulations. This interest in green chemistry urges the research community to introduce new approaches to industrial problems and take into account the biodegradability and environmental toxicology with renovated interest.^1^ On the other hand, the utilization of solvents in the chemical industry is fundamental for their many advantages, and thus, it would be difficult to eliminate them from basic operations in this industry.

The thermophysical behavior of different families of compounds obtained from biomass and classified as green solvents has been studied by our research group in the last years.^2,3^ The organic esters especially the lactates are between the chemicals studied. They can be used widely in the chemical industry applications inducing the production of less contaminant solvents. In this context, we are interested to give some approach of the thermophysical behavior of binary mixtures containing these green solvents and alcohols.

The surface tension is considered one of the most important thermophysical properties, which have an interesting contribution to research of the liquid systems and to different industrial applications, for example, separations and extractions. The study of the surface tension allows interpreting some molecular interactions of liquids.

In this paper, we present the surface behavior, trough surface tensions, and surface tension deviations of the binary mixtures involving alkyl lactates (methyl lactate and ethyl lactate) and alcohols (*n*-alkanols from methanol to 1-hexanol and isomeric butanols) at *T* = 298.15 K and at atmospheric pressure. It can be noted that we have previously studied bulk properties of some of these systems, methyl lactate and ethyl lactate with *n*-alkanols (from methanol to 1-butanol).^4^

## 2 Materials and Methods

The mass fractions of the materials were obtained using gas chromatography supplied by the suppliers, and a Karl Fischer titration was used to determine their water content using a Crison KF 1S-2B. This data is given in Table 1.

**Table 1:** Provenance and Purity of the Compounds

chemical name	CAS number	source	purity^a^(mass fraction)	water content^b^(mass fraction)
methyl lactate	547-64-8	TCI	0.997	0.000180
ethyl lactate	97-64-3	Aldrich	0.997	0.000200
methanol	67-56-1	Sigma-Aldrich	0.995	0.000165
ethanol	64-17-5	Acros	0.998	0.000145
1-propanol	71-23-8	Sigma-Aldrich	0.998	0.000195
1-butanol	71-36-3	Sigma-Aldrich	0.999	0.000175
2-butanol	78-92-2	Sigma-Aldrich	0.995	0.000190
2-methyl-1-propanol	78-83-1	Aldrich	0.998	0.000200
2-methyl-2-propanol	75-65-0	Sigma-Aldrich	0.998	0.000210
1-pentanol	71-41-0	Sigma-Aldrich	0.998	0.000270
1-hexanol	111-27-3	Sigma-Aldrich	0.993	0.000250

a As stated by the supplier by GC analysis.

b Determined by Karl Fischer titration.

The mixtures were prepared by mass using a CP225-D Sartorius Semimicro mass balance, the uncertainty being ±1 × 10^–5^ g. The corresponding uncertainty in the mole fraction is 0.001.

A Lauda TVT-2 tensiometer device was used to collect the surface tensions at the liquid–air interface, σ, of both the pure liquids and their mixtures. The densities of the liquid samples needed to calculate the corresponding surface tensions were measured using an Anton Paar DMA-5000 densimeter. The values of the densities reported in a previous paper^4^ are reproduced in Table S1 of the Supporting Information. The temperature was maintained constant within ±0.01 K by means of a Lauda E-200 thermostat. For each surface tension measurement, 50 volume drops were determined and averaged. The uncertainties of surface tension values are affected by the value of temperature and density difference. The combined expanded uncertainty for surface tension is 0.2 mN·m^–1^.

## 3 Results and Discussion

In Table 2, the experimental surface tensions of the pure compounds at *T* = 298.15 K and at *p* = 0.1 MPa are given. For comparison, surface tensions obtained from the literature^3,5−15^ have been also included in this table. The agreement between both data sets is excellent.

**Table 2:** Surface Tensions, σ, of Pure Compounds at *T* = 298.15 K and at *p* = 0.1 MPa and Comparison with the Literature Data^a^

	σ/(mN·m^–1^)	
Compound	exptl.	lit.
methyl lactate	32.84	32.90^3^
ethyl lactate	29.47	29.49^3^
methanol	22.19	22.14^5^ 22.19^6^
ethanol	21.90	21.86^7^ 21.9^8^
1-propanol	23.37	23.34^7^ 23.31^9^
1-butanol	24.28	24.20^10^ 24.18^11^
2-butanol	23.13	23.46^11^ 23.00^12^
2-methyl-1-propanol	22.38	22.30^11^ 22.44^12^
2-methyl-2-propanol	20.24	20.13^11^ 20.11^12^
1-pentanol	25.28	25.29^9^ 25.36^13^
1-hexanol	25.77	25.79^14^ 25.81^15^

a Standard uncertainties *u* are *u*(*T*) = 0.01 K and *u*(*p*) = 0.0025 MPa, and the combined expanded uncertainties *U*_c_ are *U*_c_(σ) = 0.2 mN·m^–1^ with a 0.95 level of confidence (*k* = 2).

**Figure 1:**
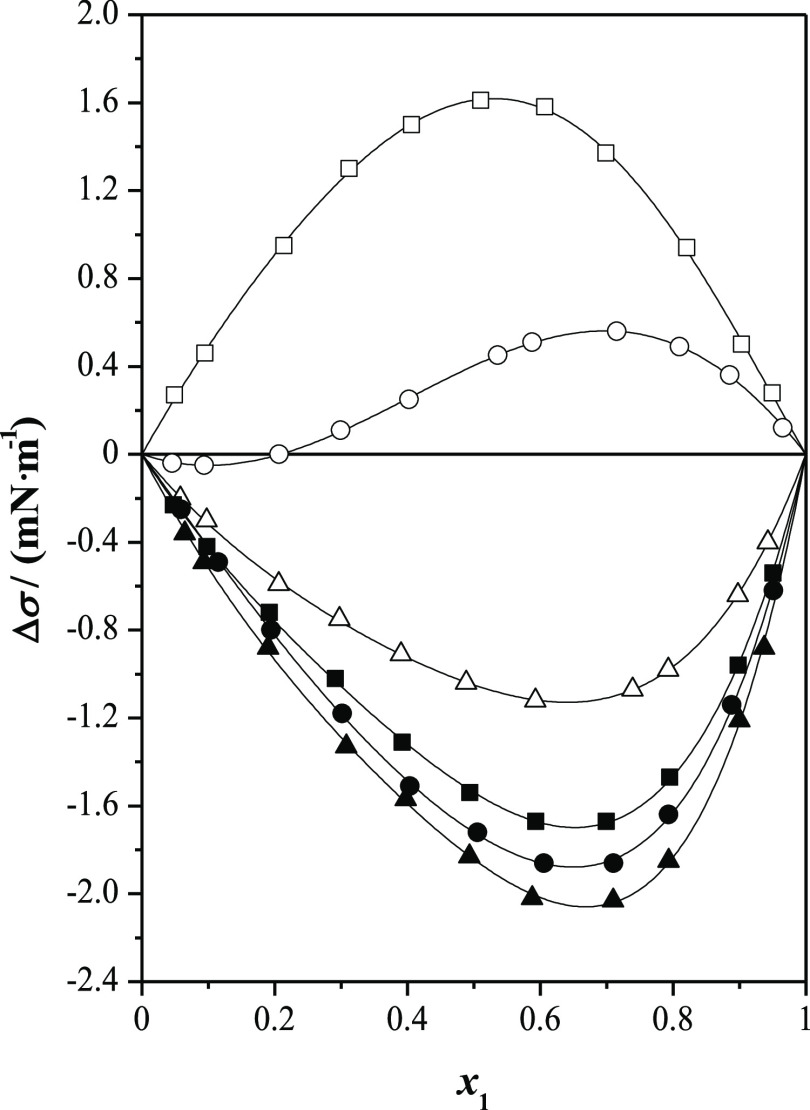
Surface tension deviations, Δσ, for methyl lactate (1) + *n*-alkanol (2) at *T* = 298.15 K and at *p* = 0.1 MPa as a function of the mole fraction, *x*_1_: (□) methanol; (◯) ethanol; (△) 1-propanol; (■) 1-butanol; (●)1-pentanol; (▲) 1-hexanol; and (—) Myers–Scott equation.

**Figure 2:**
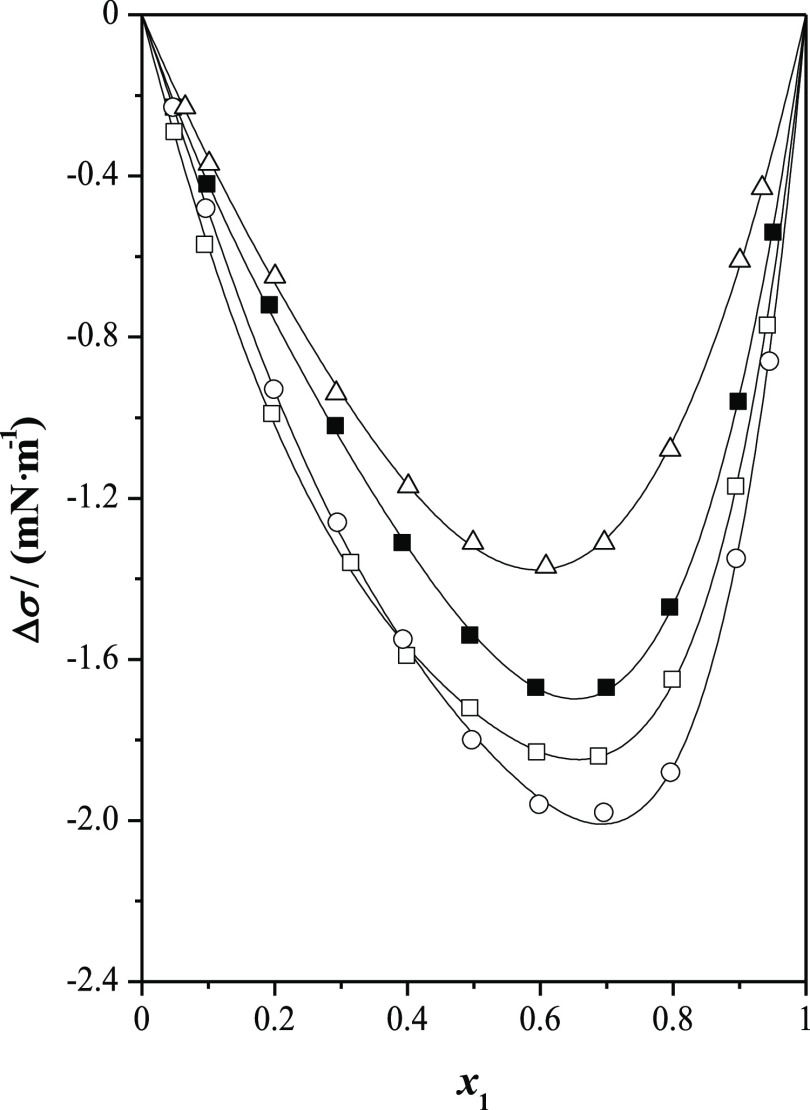
Surface tension deviations, Δσ, for methyl lactate (1) + isomeric butanol (2) at *T* = 298.15 K and at *p* = 0.1 MPa as a function of the mole fraction, *x*_1_: (■) l-butanol; (□) 2-butanol; (◯) 2-methyl-1-propanol; (△) 2-methyl-2-propanol; and (—) Myers–Scott equation.

**Figure 3:**
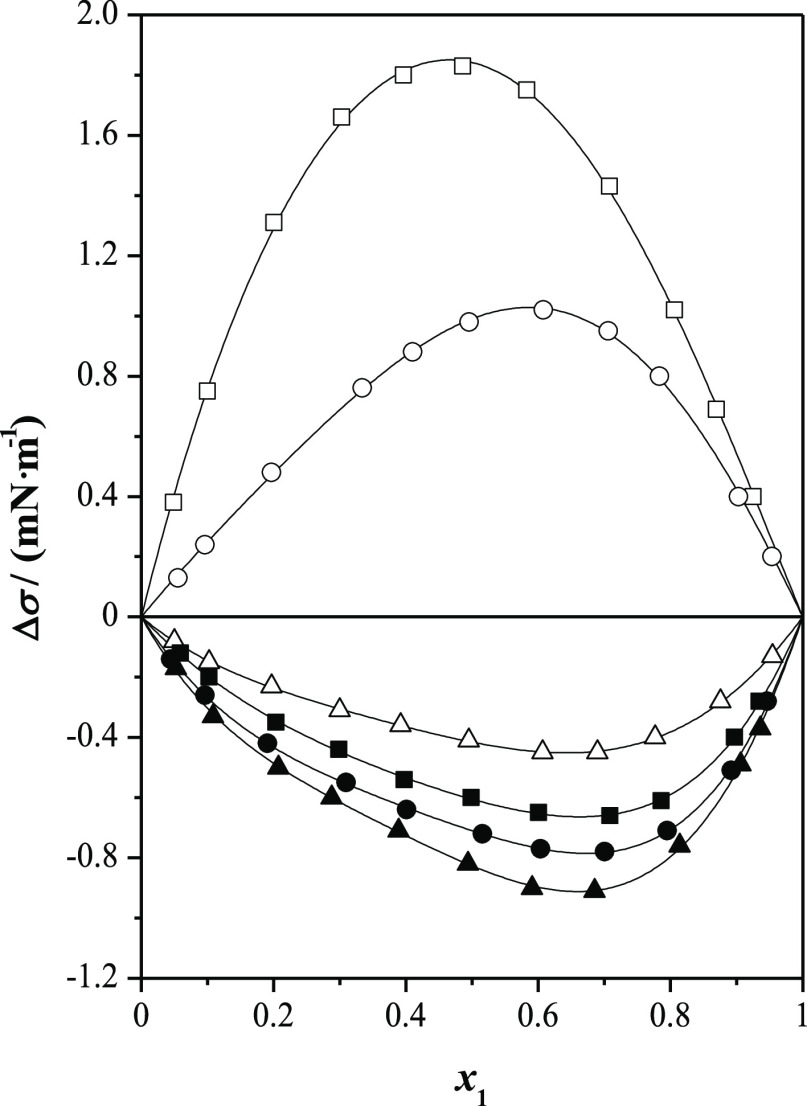
Surface tension deviations, Δσ, for ethyl lactate (1) + *n*-alkanol (2) at *T* = 298.15 K and at *p* = 0.1 MPa as a function of the mole fraction, *x*_1_: (□) methanol; (◯) ethanol; (△) 1-propanol; (■) 1-butanol; (●)1-pentanol; (▲) 1-hexanol; and (—) Myers–Scott equation.

**Figure 4:**
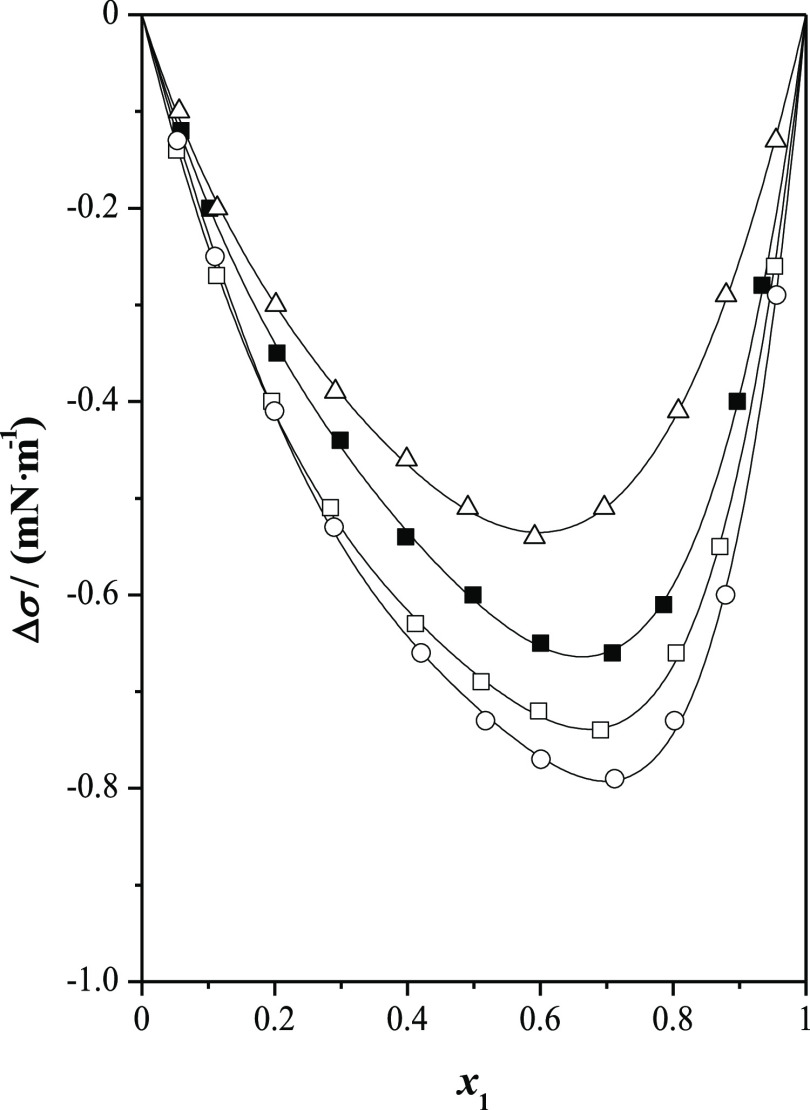
Surface tension deviations, Δσ, for ethyl lactate (1) + isomeric butanol (2) at *T* = 298.15 K and at *p* = 0.1 MPa as a function of the mole fraction, *x*_1_: (■) l-butanol; (□) 2-butanol; (◯) 2-methyl-1-propanol; (△) 2-methyl-2-propanol; and (—) Myers–Scott equation.

In Table 3, the surface tensions, σ, and surface tension deviations, Δσ, of the binary mixtures are collected. The surface tension deviations as a function of the mole fraction are graphically represented in Figures 1, 2, 3, and 4.

**Table 3:** Surface Tensions, σ, and Surface Tension Deviations, Δσ, for the Binary Mixtures Alkyl Lactate (1) + Alkanol (2) at *T* = 298.15 K and at *p* = 0.1 MPa^a^

*x*_1_	σ/(mN·m^–1^)	Δσ/(mN·m^–1^)	*x*_1_	σ/(mN·m^–1^)	Δσ/(mN·m^–1^)
Methyl Lactate (1) + Methanol (2)					
0.0000	22.19		0.6069	30.23	1.58
0.0495	22.99	0.27	0.6987	31.00	1.37
0.0950	23.66	0.46	0.8209	31.87	0.94
0.2139	25.42	0.95	0.9029	32.31	0.50
0.3121	26.81	1.30	0.9491	32.58	0.28
0.4063	28.02	1.50	1.0000	32.84	
0.5106	29.24	1.61			
Methyl Lactate (1) + Ethanol (2)					
0.0000	21.90		0.5878	28.84	0.51
0.0455	22.36	–0.04	0.7152	30.28	0.56
0.0940	22.88	–0.05	0.8096	31.25	0.49
0.2066	24.16	0.00	0.8851	31.94	0.36
0.2995	25.29	0.11	0.9653	32.58	0.12
0.4027	26.56	0.25	1.0000	32.84	
0.5361	28.21	0.45			
Methyl Lactate (1) + 1-Propanol (2)					
0.0000	23.37		0.5928	27.86	–1.12
0.0582	23.72	–0.20	0.7390	29.30	–1.07
0.0975	23.99	–0.30	0.7925	29.89	–0.98
0.2058	24.73	–0.59	0.8977	31.23	–0.64
0.2977	25.44	–0.75	0.9424	31.89	–0.40
0.3904	26.16	–0.91	1.0000	32.84	
0.4884	26.96	–1.04			
Methyl Lactate (1) + 1-Butanol (2)					
0.0000	24.28		0.5932	27.69	–1.67
0.0473	24.45	–0.23	0.6994	28.60	–1.67
0.0979	24.70	–0.42	0.7952	29.62	–1.47
0.1920	25.20	–0.72	0.8984	31.01	–0.96
0.2915	25.76	–1.02	0.9507	31.88	–0.54
0.3920	26.33	–1.31	1.0000	32.84	
0.4939	26.97	–1.54			
Methyl Lactate (1) + 2-Butanol (2)					
0.0000	23.13		0.5945	27.07	–1.83
0.0488	23.31	–0.29	0.6878	27.97	–1.84
0.0945	23.48	–0.57	0.7990	29.24	–1.65
0.1958	24.04	–0.99	0.8945	30.65	–1.17
0.3147	24.83	–1.36	0.9421	31.51	–0.77
0.3987	25.41	–1.59	1.0000	32.84	
0.4937	26.20	–1.72			
Methyl Lactate (1) + 2-Methyl-1-Propanol (2)					
0.0000	22.38		0.5980	26.68	–1.96
0.0470	22.64	–0.23	0.6960	27.68	–1.98
0.0962	22.91	–0.48	0.7961	28.83	–1.88
0.1989	23.53	–0.93	0.8949	30.39	–1.35
0.2945	24.20	–1.26	0.9451	31.41	–0.86
0.3930	24.94	–1.55	1.0000	32.84	
0.4969	25.78	–1.80			
Methyl Lactate (1) + 2-Methyl-2-Propanol (2)					
0.0000	20.24		0.6084	26.54	–1.37
0.0651	20.83	–0.23	0.6964	27.70	–1.31
0.1014	21.15	–0.37	0.7958	29.19	–1.08
0.2009	22.12	–0.65	0.9008	30.98	–0.61
0.2926	22.99	–0.94	0.9342	31.58	–0.43
0.4011	24.12	–1.17	1.0000	32.84	
0.4987	25.21	–1.31			
Methyl Lactate (1) + 1-Pentanol (2)					
0.0000	25.28		0.6055	28.00	–1.86
0.0591	25.48	–0.25	0.7101	28.79	–1.86
0.1153	25.66	–0.49	0.7935	29.64	–1.64
0.1947	25.95	–0.80	0.8885	30.86	–1.14
0.3019	26.38	–1.18	0.9511	31.85	–0.62
0.4034	26.82	–1.51	1.0000	32.84	
0.5052	27.38	–1.72			
Methyl Lactate (1) + 1-Hexanol (2)					
0.0000	25.77		0.5876	27.90	–2.02
0.0646	25.87	–0.36	0.7100	28.76	–2.03
0.0931	25.94	–0.49	0.7932	29.53	–1.85
0.1905	26.24	–0.88	0.8998	30.92	–1.21
0.3082	26.62	–1.33	0.9372	31.52	–0.88
0.3978	27.01	–1.57	1.0000	32.84	
0.4934	27.43	–1.83			
Ethyl Lactate (1) + Methanol (2)					
0.0000	22.19		0.5829	28.18	1.75
0.0490	22.93	0.38	0.7081	28.77	1.43
0.1001	23.67	0.75	0.8061	29.08	1.02
0.2005	24.96	1.31	0.8694	29.21	0.69
0.3031	26.06	1.66	0.9252	29.33	0.40
0.3966	26.88	1.80	1.0000	29.47	
0.4860	27.56	1.83			
Ethyl Lactate (1) + Ethanol (2)					
0.0000	21.90		0.6080	27.52	1.02
0.0557	22.45	0.13	0.7064	28.20	0.95
0.0966	22.87	0.24	0.7838	28.63	0.80
0.1971	23.87	0.48	0.9033	29.14	0.40
0.3344	25.19	0.76	0.9537	29.32	0.20
0.4102	25.89	0.88	1.0000	29.47	
0.4957	26.63	0.98			
Ethyl Lactate (1) + 1-Propanol (2)					
0.0000	23.37		0.6067	26.62	–0.45
0.0498	23.59	–0.08	0.6895	27.13	–0.45
0.1026	23.85	–0.15	0.7771	27.71	–0.40
0.1970	24.34	–0.23	0.8759	28.43	–0.28
0.2999	24.89	–0.31	0.9540	29.06	–0.13
0.3921	25.40	–0.36	1.0000	29.47	
0.4954	25.98	–0.41			
Ethyl Lactate (1) + 1-Butanol (2)					
0.0000	24.28		0.6006	26.75	–0.65
0.0591	24.47	–0.12	0.7084	27.30	–0.66
0.1027	24.61	–0.20	0.7861	27.75	–0.61
0.2037	24.99	–0.35	0.8972	28.54	–0.40
0.2987	25.39	–0.44	0.9344	28.85	–0.28
0.3974	25.80	–0.54	1.0000	29.47	
0.4989	26.27	–0.60			
Ethyl Lactate (1) + 2-Butanol (2)					
0.0000	23.13		0.5975	26.20	–0.72
0.0525	23.32	–0.14	0.6908	26.77	–0.74
0.1127	23.57	–0.27	0.8048	27.57	–0.66
0.1957	23.97	–0.40	0.8710	28.10	–0.55
0.2842	24.42	–0.51	0.9533	28.91	–0.26
0.4119	25.11	–0.63	1.0000	29.47	
0.5107	25.68	–0.69			
Ethyl Lactate (1) + 2-Methyl-1-Propanol (2)					
0.0000	22.38		0.6012	25.87	–0.77
0.0535	22.63	–0.13	0.7124	26.64	–0.79
0.1101	22.91	–0.25	0.8023	27.34	–0.73
0.1998	23.39	–0.41	0.8795	28.02	–0.60
0.2897	23.90	–0.53	0.9562	28.87	–0.29
0.4209	24.70	–0.66	1.0000	29.47	
0.5175	25.32	–0.73			
Ethyl Lactate (1) + 2-Methyl-2-Propanol (2)					
0.0000	20.24		0.5915	25.16	–0.54
0.0563	20.66	–0.10	0.6964	26.16	–0.51
0.1137	21.09	–0.20	0.8080	27.29	–0.41
0.2021	21.81	–0.30	0.8801	28.07	–0.29
0.2916	22.54	–0.39	0.9547	28.92	–0.13
0.3988	23.46	–0.46	1.0000	29.47	
0.4908	24.26	–0.51			
Ethyl Lactate (1) + 1-Pentanol (2)					
0.0000	25.28		0.6036	27.04	–0.77
0.0443	25.33	–0.14	0.7007	27.44	–0.78
0.0962	25.42	–0.26	0.7953	27.90	–0.71
0.1911	25.66	–0.42	0.8922	28.51	–0.51
0.3100	26.03	–0.55	0.9465	28.97	–0.28
0.4013	26.32	–0.64	1.0000	29.47	
0.5159	26.72	–0.72			
Ethyl Lactate (1) + 1-Hexanol (2)					
0.0000	25.77		0.5906	27.06	–0.90
0.0524	25.79	–0.17	0.6852	27.40	–0.91
0.1088	25.84	–0.33	0.8142	28.02	–0.76
0.2074	26.04	–0.50	0.9060	28.63	–0.49
0.2877	26.23	–0.60	0.9359	28.86	–0.37
0.3893	26.50	–0.71	1.0000	29.47	
0.4938	26.78	–0.82			

a Standard uncertainties *u* are *u*(*T*) = 0.01 K, *u*(*p*) = 0.0025 MPa, and *u*(*x*_1_) = 0.001, and the combined expanded uncertainties *U*_c_ are *U*_c_(σ) = 0.2 mN·m^–1^ with a 0.95 level of confidence (*k* = 2).

The surface tension deviation with respect to a linear dependence on the mole fraction, Δσ, was determined using the following equation:
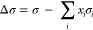
1where σ, σ_*i*_, and *x*_*i*_ are the surface tension of the mixture, the surface tension of component *i*, and the mole fraction of component *i*, respectively.

For each binary mixture, the surface tension deviation was correlated with the molar fraction by means of the Myers–Scott equation^16^
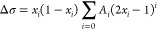
2the *A*_*i*_ coefficients were determined by the least squares method, and the number of these coefficients was selected to minimize the standard deviation for the fit. These parameters, *A*_*i*_, along with the standard deviation, σ(Δσ), are summarized in Table 4.

**Table 4:** Coefficients and Standard Deviations, σ(Δσ), for the Myers–Scott Equation

System	*A*_0_	*A*_1_	*A*_2_	*A*_3_	σ(Δσ)
Methyl Lactate +					
Methanol	6.45	0.93	–1.18	–1.01	0.02
ethanol	1.61	2.77	–0.17	–0.31	0.01
1-propanol	–4.20	–2.03	–1.63	–0.18	0.01
1-butanol	–6.15	–3.71	–2.21	0.11	0.01
2-butanol	–6.92	–2.63	–4.01	–1.93	0.02
2-methyl-1-propanol	–7.15	–3.76	–4.44	–3.05	0.02
2-methyl-2-propanol	–5.29	–2.23	–0.24	0.55	0.01
1-pentanol	–6.88	–3.84	–2.17	–0.99	0.02
1-hexanol	–7.37	–4.41	–3.56	–0.74	0.02
Ethyl Lactate +					
Methanol	7.37	–1.04	–0.23	–0.72	0.02
Ethanol	3.96	1.69	–0.36	–0.77	0.01
1-propanol	–1.66	–0.87	–0.75	0.31	0.04
1-butanol	–2.42	–1.23	–1.34	–0.22	0.06
2-butanol	–2.72	–1.12	–1.86	–0.68	0.01
2-methyl-1-propanol	–2.85	–1.24	–2.10	–1.34	0.01
2-methyl-2-propanol	–2.06	–0.74	–0.50	0.31	0.01
1-pentanol	–2.84	–1.42	–2.01		0.01
1-hexanol	–3.31	–1.80	–1.94	0.52	0.01

In Figure 1, the surface tension deviations for methyl lactate (1) + *n*-alkanol (2) systems are presented. These surface tension deviations show three different behaviors. We can note that the methyl lactate + methanol system shows positive deviations over the whole composition range, with a maximum found at around *x*_1_ = 0.5. However, Δσ values exhibit a sigmoid shape for the methyl lactate + ethanol mixture. Being the surface tension deviation negative in the lactate- poor region and becomes positive when *x*_1_ reaches 0.2. Finally, the mixtures methyl lactate + *n*-alkanol (1-propanol to 1-hexanol) show negative deviations. The minimum of the surface tension deviation occurs in the lactate-rich region (0.65 < *x*_1_ < 0.7). Δσ values become more negative from 1-propanol to 1-hexanol.

Figure 2 gives the surface tension deviations obtained for methyl lactate (1) + isomeric butanol (2) mixtures. In this case, surface tension deviations present negative values. The minima take place in the lactate-rich region (0.65 < *x*_1_ < 0.75). Δσ values are more negative in the following succeeding order: 2-methyl-2-propanol < 1-butanol < 2-butanol < 2-methyl-1-propanol.

In Figure 3, the surface tension deviations for ethyl lactate (1) + *n*-alkanol (2) are plotted. The surface tension deviations show two different behaviors. Positive deviation is observed for the mixtures involving methanol and ethanol. The maxima occur at *x*_1_ = 0.5 and *x*_1_ = 0.6 for methanol and ethanol, respectively, while for the rest of *n*-alkanol (1-propanol to 1-hexanol), negative deviation is obtained over the whole composition range. The minima occur at *x*_1_ = 0.65. The following order gives more negative surface tension deviations: 1-propanol < 1-butanol < 1-pentanol < 1-hexanol.

Finally, Figure 4 represents the surface tension deviation for ethyl lactate (1) + isomeric butanol (2) mixtures. All these systems show negative deviations. The minima occur in the (0.5 < *x*_1_ < 0.7) region. The following order gives more negative Δσ values: 2-methyl-2-propanol < 1-butanol < 2-butanol < 2-methyl-1-propanol.

It can be noted that the influence of the butanol ramification on the surface behavior is less marked than the length of the *n*-alkanol chain.

On the other hand, the binary mixtures containing ethyl lactate show bigger positive surface tension deviations or less negative surface tension deviations than the systems with methyl lactate.

The surface tension is a thermophysical property related to both molecular interactions and structural factors between the mixed components. The surface tensions of alkyl lactates are bigger than the surface tensions of the alkanols, showing that the cohesive interactions among alkyl lactate molecules are stronger than those among the alkanol ones. The positive surface tensions obtained for the systems containing methanol and ethanol could be explained by strong interactions between alkyl lactates and methanol and ethanol. This heteroassociation maintains the methanol and ethanol molecules in the bulk, preventing their migration from the bulk to the surface and leading to positive Δσ values. For the rest of alkanols, the interactions between the components in the mixing process are lower than the weakening of self-interactions in the pure liquids. Therefore, the compounds of lower surface tension, alkanols, experience surface migration, leading to negative surface tension deviations.^17^ With respect to structural factors, they are more related to the volumetric behavior.

**Figure 5:**
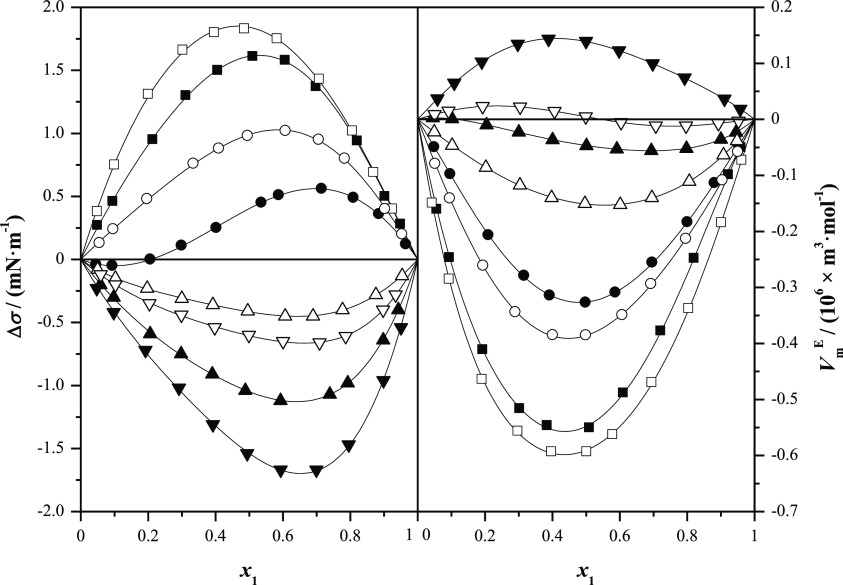
Comparison of surface tension deviations, Δσ, with excess molar volumes, , for alkyl lactates (1) + *n*-alkanol (2) at *T* = 298.15 K and at *p* = 0.1 MPa as a function of the mole fraction, *x*_1_: full symbols, methyl lactate; open symbols, ethyl lactate; ■, methanol; ●, ethanol; ▲, 1-propanol; and ▼, 1-butanol.

**Figure 6:**
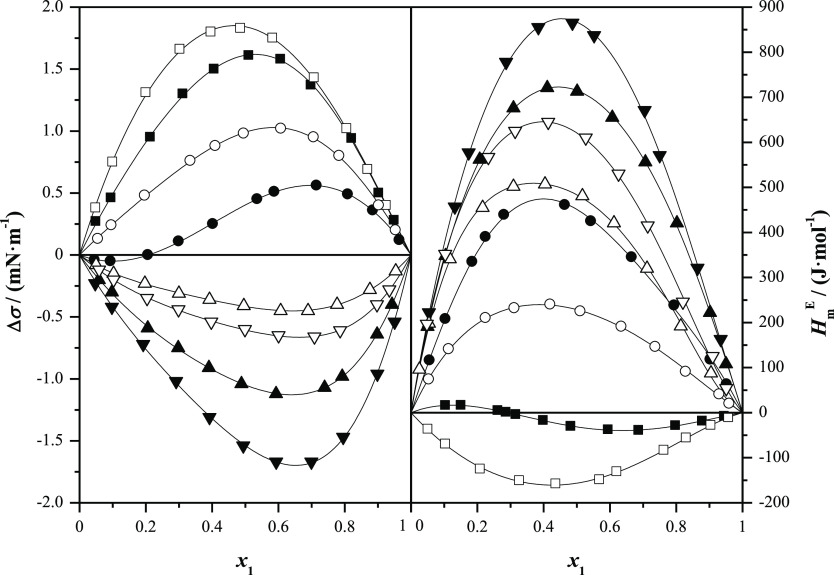
Comparison of surface tension deviations, Δσ, with excess molar enthalpies, , for alkyl lactates (1) + *n*-alkanol (2) at *T* = 298.15 K and at *p* = 0.1 MPa as a function of the mole fraction, *x*_1_: full symbols, methyl lactate; open symbols, ethyl lactate; ■, methanol; ●, ethanol; ▲, 1-propanol; and ▼, 1-butanol.

In this sense, Figures 5 and 6 present a graphical comparison of surface tension deviations with both excess molar volumes and excess molar enthalpies, previously published^4^ (systems methyl lactate and ethyl lactate with *n*-alkanols at *T* = 298.15 K and at *p* = 0.1 MPa). The excess molar volumes depend on both the strength of the molecular interactions and structural effects. For the systems alkyl lactate with *n*-alkanol,  are negative, except for the systems containing 1-butanol, although for the mixture ethyl lactate + 1-butanol, the excess molar volumes show a sigmoidal behavior with the composition. On the other hand, excess molar volumes decrease as the length of the alcohol decreases. This volumetric behavior shows that a compact packing occurs between the mixed compounds, reducing the free volume among molecules. On the contrary, the surface tension deviations for both esters decrease with the length of the alcohol. Showing, these properties excess molar volumes and surface tension deviations an opposite behavior. Regarding calorimetric behavior, it mainly depends on molecular interactions. The  for these systems are clearly positive, except for the systems containing methanol; in the case of methyl lactate, excess molar enthalpies show a sigmoidal behavior, while for ethyl lactate, their values are negative in the whole composition range. The excess molar enthalpies increase with the length of the alkanol. These positive values show that the interactions between unlike molecules are less favorable than self-interactions in the pure compounds, especially in the longer *n*-alkanols, while for methanol and ethanol, some heteroassociation takes place, lowering the excess molar enthalpy. That is, something similar to the comparison between volumetric and surface behavior occurs with calorimetric and surface behavior.^18^

## 4 Summary

In this contribution, the surface tension of binary mixtures involving alkyl lactates (methyl lactate and ethyl lactate) and alcohols (*n*-alkanols from methanol to 1-hexanol and isomeric butanols) is presented at *T* = 298.15 K and at *p* = 0.1 MPa.

Using a CP225-D Sartorius Semimicro mass balance, the mixtures were prepared. Their surface tensions were determined by the drop volume method using a Lauda TVT-2 tensiometer. For the calculation of the surface tension, the densities of the samples are needed, and they were measured by means of an Anton Paar DMA-5000 densimeter.

The surface tension deviation for all the mixtures was calculated. For the mixtures alkyl lactate with methanol and ethanol, the surface tension deviations are positive, although in the case of the ethyl lactate + ethanol system, Δσ presents slightly negative values in the lactate-poor region. For the rest of the mixtures, the surface tension deviations are negative. For *n*-alkanols, Δσ becomes more negative with the length of *n*-alkanol, and for the isomeric butanols, the following order gives more negative surface tension deviations: 2-methyl-2-propanol < 1-butanol < 2-butanol < 2-methyl-1-propanol.

## Supporting Information Available

Densities, ρ, as the function of the mole fraction, *x*_1_, of the binary mixtures alkyl lactate (1) + alkanol (2) at *T* = 298.15 K and at *p* = 0.1 MPa (PDF)
