# Supplementary material for: Surface Tensions of Alkyl Lactates with n-Alkanols or Branched Alkanols
Source: J Chem Eng Data. 2022 Dec 28;68(1):100–7. doi: 10.1021/acs.jced.2c00629 (PMC9844212; doi:10.1021/acs.jced.2c00629)
Supplement: Supplementary file 1 — je2c00629_si_001.pdf [file je2c00629_si_001.pdf]

SUPPLEMENTARY INFORMATION

Surface Tensions of Alkyl Lactates with n-Alkanols or Branched Alkanols

Carmen Almodovar[‡], Héctor Artigas[‡], Saoussen Wacharine[†], Kaïs Antar[†], Carlos Lafuente[‡,*]

[‡] Departamento de Química Física, Facultad de Ciencias, Universidad de Zaragoza, 50009 Zaragoza, Spain

[†] Université de Tunis EL Manar, Faculté des Sciences, Laboratoire des Matériaux, Cristallochimie et Thermodynamique Appliquée, LR15ES01, Département de Chimie, 2092 Tunis, Tunisia

*Corresponding author. E-mail address: celadi@unizar.es

TABLE S1. Densities, ρ, as function of the mole fraction, x_1, of the binary mixtures alkyl lactate (1) + alkanol (2) at T = 298.15 K and at p = 0.1 MPa.[a]

x_1	ρ / (g·cm^{-3})	x_1	ρ / (g·cm^{-3})	x_1	ρ / (g·cm^{-3})
		Methyl lactate (1) + methanol (2)			
0.0000	0.78653	0.3844	0.97404	0.8206	1.06482
0.0565	0.82667	0.5099	1.00810	0.9225	1.07813
0.0945	0.85035	0.6105	1.02988	0.9601	1.08270
0.1933	0.90212	0.7215	1.04984	1.0000	1.08735
0.3029	0.94703				
		Methyl lactate (1) + ethanol (2)			
0.0000	0.78516	0.3921	0.94417	0.8007	1.04953
0.0492	0.80935	0.4987	0.97629	0.8814	1.06565
0.0968	0.83145	0.5872	1.00020	0.9535	1.07911
0.2099	0.87928	0.6963	1.02672	1.0000	1.08735
0.3161	0.91872				
		Methyl lactate (1) + 1-propanol (2)			
0.0000	0.79966	0.4004	0.93231	0.7978	1.04020
0.0450	0.81593	0.5012	0.96171	0.9000	1.06467
0.1025	0.83620	0.5911	0.98671	0.9450	1.07504
0.2047	0.87081	0.6935	1.01388	1.0000	1.08735
0.3000	0.90152				
		Methyl lactate (1) + 1-butanol (2)			
0.0000	0.80595	0.3887	0.91662	0.7926	1.03002
0.0589	0.82283	0.4999	0.94804	0.9107	1.06272
0.1088	0.83709	0.5977	0.97558	0.9577	1.07569
0.1905	0.86037	0.6976	1.00357	1.0000	1.08735
0.2973	0.89071				
		Ethyl lactate (1) + methanol (2)			
0.0000	0.78653	0.3976	0.95194	0.8045	1.01266
0.0432	0.81663	0.5018	0.97285	0.9018	1.02120
0.0935	0.84609	0.5789	0.98546	0.9622	1.02579
0.1911	0.89078	0.6950	1.00096	1.0000	1.02845
0.2979	0.92651				
		Ethyl lactate (1) + ethanol (2)			
0.0000	0.78516	0.4011	0.92758	0.7965	1.00243
0.0522	0.80988	0.4995	0.95030	0.9047	1.01703
0.0957	0.82874	0.6076	0.97175	0.9511	1.02275
0.1946	0.86653	0.6931	0.98654	1.0000	1.02845
0.2902	0.89743				

TABLE S1. Continuation.

x_1	ρ / (g·cm^{-3})	x_1	ρ / (g·cm^{-3})	x_1	ρ / (g·cm^{-3})
		Ethyl lactate (1) + 1-propanol (2)			
0.0000	0.79966	0.3973	0.91589	0.8061	0.99837
0.0489	0.81657	0.4963	0.93864	0.9076	1.01474
0.1025	0.83415	0.6012	0.96068	0.9482	1.02090
0.2027	0.86458	0.6951	0.97876	1.0000	1.02845
0.3036	0.89235				
		Ethyl lactate (1) + 1-butanol (2)			
0.0000	0.80595	0.4061	0.90831	0.7968	0.99082
0.0502	0.81965	0.4991	0.92927	0.8927	1.00898
0.0914	0.83065	0.6037	0.95184	0.9527	1.01997
0.1929	0.85689	0.7070	0.97311	1.0000	1.02845
0.3041	0.88431				

[a] Previously published (Sanchez, J. ; Artigas, H. ; Gascón, I. ; Lafuente, C. Thermodynamic behaviour of alkyl lactate-alkanol systems *J. Chem. Thermodyn.* **2018,** 127, 33-38.)
